# Gastrointestinal Stromal Tumors Associated with Neurofibromatosis 1: A Single Centre Experience and Systematic Review of the Literature Including 252 Cases

**DOI:** 10.1155/2013/398570

**Published:** 2013-12-09

**Authors:** Pier Federico Salvi, Laura Lorenzon, Salvatore Caterino, Laura Antolino, Maria Serena Antonelli, Genoveffa Balducci

**Affiliations:** Surgical and Medical Department of Translational Medicine, Sant'Andrea Hospital, Faculty of Medicine and Psychology, University of Rome La Sapienza, St. Andrea Hospital, Via di Grottarossa 1035-39, 00189 Rome, Italy

## Abstract

*Aims*. The objectives of this study were (a) to report our experience regarding the association between neurofibromatosis type 1 (NF1) and gastrointestinal stromal tumors (GISTs); (b) to provide a systematic review of the literature in this field; and (c) to compare the features of NF1-associated GISTs with those reported in sporadic GISTs. *Methods*. We reported two cases of NF1-associated GISTs. Moreover we reviewed 23 case reports/series including 252 GISTs detected in 126 NF1 patients; the data obtained from different studies were analyzed and compared to those of the sporadic GISTs undergone surgical treatment at our centre. *Results*. NF1 patients presenting with GISTs had a homogeneous M/F ratio with a mean age of 52.8 years. NF1-associated GISTs were often reported as multiple tumors, mainly incidental, localized at the jejunum, with a mean diameter of 3.8 cm, a mean mitotic count of 3.0/50 HPF, and KIT/PDGFR**α** wild type. We reported a statistical difference comparing the age and the symptoms at presentation, the tumors' diameters and localizations, and the risk criteria of the NF1-associated GISTs comparing to those documented in sporadic GISTs. *Conclusions*. NF1-associated GISTs seem to have a distinct phenotype, specifically younger age, distal localization, small diameter, and absence of KIT/PDGRF**α** mutations.

## 1. Introduction

Neurofibromatosis type 1 (NF1, von Recklinghausen's disease) is an autosomal-dominant disorder occurring in 1 out of 3,000 births that is caused by the inactivation of the NF1 gene. NF1 is a tumor suppressor that encodes for the neurofibromin protein, a member of the Ras family. The inactivation might be a familial condition with an autosomal-dominant inheritance pattern; otherwise it might be sporadic [1, 2].

The disease is characterized by cutaneous neurofibromas, café au lait macules, axillary and inguinal freckling, and Lisch nodules.

NF1 is also associated with several tumors, including tumors of the nervous system (central and peripheral) and of the gastrointestinal (GI) tract, with the gastrointestinal stromal tumors (GISTs) indicated as the most common GI NF1-associated tumors [3].

GISTs are mesenchymal and usually kit positive tumors, originating from the interstitial cell of Cajal or their related stem cells [4, 5]. The incidence of the GISTs has been reported in 10–20 new cases per million/year [6]. GISTs represent 80% of mesenchymal GI tumors and 0.1–3% of all GI malignancies [7–10]. GIST's pathogenesis is related to kit and platelet-derived growth factor receptor alpha (PDGFRa) mutation. Kit and PDGFRa encode for similar type III receptor tyrosine kinase proteins: these mutations are somatic and occur only in the neoplastic tissue of sporadic GISTs, whereas constitutional mutations in familial GISTs occur in every cell of the body and are inheritable [11–14].

Over the last few years several case reports documented the association between GISTs and NF1 syndrome; however to date there is a lack of reviews in this field with the objective of describing the clinical and pathological features of GISTs presenting in NF1 patients.

Aims of this study were (a) to report our experience regarding this association; (b) to provide a systematic review of the literature in this field including 252 GISTs described in 126 NF1 patients with the objective of analyzing the clinical, pathological, and molecular features of these tumors in patients affected by this condition; and (c) to compare the clinical/pathological features of GISTs presenting associated with NF1 with those reported in sporadic GISTs.

## 2. Materials and Methods

### 2.1. Systematic Review: Data Source and Search Strategies

**Figure 1:**
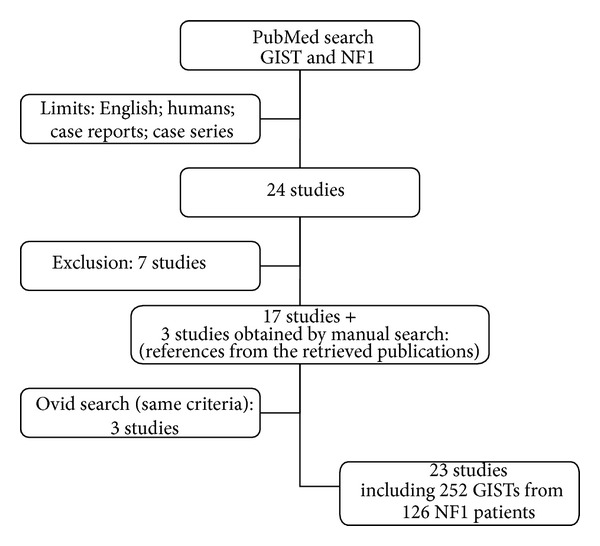
Systematic review: study design according to the PRISMA statement.

This investigation has been conducted adhering at the PRISMA statements for review and meta-analysis (Figure 1). We conducted a systematic review of the literature by searching PubMed database for all published series and case reports investigating the association of NF1 and GIST (Keywords: “GIST and NF1”, language: English; filter “human” studies); the Medline search was conducted at the beginning of September 2013 and retrieved 24 papers. We also included references from the retrieved publications (n 3 manuscripts).

Published series with the aim to investigate exclusively the molecular profile (n 1 study), the NF1 radiological features (n 1 study), or its association with other gastrointestinal tumors, for example, gastrointestinal schwannomas (n 1 article), were excluded.

Moreover we also excluded those reviews in this field that did not include patients' presentation (n 2 studies), one paper that reported GISTs without signs of NF1 syndrome and another study investigating the intestinal neurofibromatosis.

The same search strategies have been applied to the Ovid database and provided the addition of 3 more papers (after the manual removal of duplicate references).

Overall the systematic review has been conducted on 23 articles published from 2004 to date [15–37], plus the two patients we herein presented.

Authors conducting this review of the literature were blinded to authors' and journals' names and did not consider any journal's score (e.g., journal's Impact Factors) of the published case/series as an exclusion criteria for this study.

We collected data regarding study populations, number of investigated patients and GISTs, familiar or sporadic history of NF1, age at presentation of the GISTs, symptoms, sex of the patients, tumors' location, tumors' diameter, and number of mitoses. The morphologic appearance and cellular descriptions (generally referred to as epithelioid, spindle, and mixed cells) were also recorded along with the immunohistochemistry for CD117 (c-kit), S-100 protein, CD-34, and SMA-alpha. Whenever available, we included data regarding GIST risk classification and the studies investigating c-kit and PDGFRa mutations.

### 2.2. Statistical Analysis

With respect to the systematic review, we pooled together the data obtained from different studies in order to analyze a large series of patients and of clinical and pathological variables. Patients' clinical features and tumors' pathological records were thus analysed using means and standard deviations for quantitative variables and using frequencies and percents for categorical variables.

Moreover, the clinical and pathological features of the GISTs presenting associated with the NF1 syndrome (including age at presentation, M/F ratio, symptoms, tumors' diameter and localization, and risk criteria) retrieved from the international literature have been compared for statistical purpose to those obtained in a personal case series of sporadic GISTs undergone surgical resection at our department. Variables were compared using the *t*-test for continuous variable and the Chi-square test for categorical variables; all test were two-tailed and a *P* < 0.05 was considered of statistical significance value. All statistical evaluations were conducted with the statistical software MedCalc version 11.4.4.0.

## 3. Results

### 3.1. Personal Cases Series Presentation

**Case 1**

A 71-year-old male patient with a history of NF1 presented with an abdominal mass incidentally detected in the work-up of an abdominal aortic aneurism.

**Figure 2:**
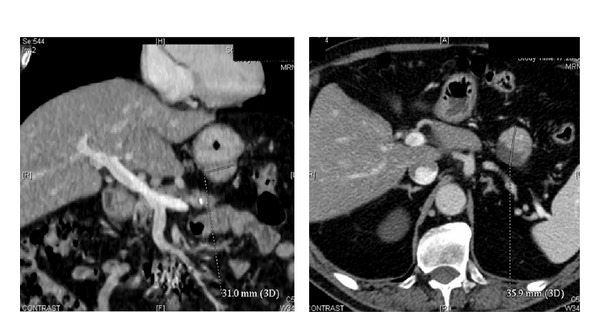
Abdominal CT scan documenting a 3.6 cm GIST of the stomach.

The patient's past medical history was consistent with hypertension and cholecystectomy for lithiasis. The abdominal CT scan documented a gastric mass of 3.6 cm (Figure 2); the patient underwent a gastroscopy that documented an atrophic gastritis. The patient was scheduled for a surgical procedure and a laparoscopic wedge resection of the posterior gastric wall was performed. The postoperative course was uneventful and the patient was discharged on 6th postoperative day.

The pathological examination documented a gastric GIST (c-KIT+, DOG1+) with spindle cell morphology, with a mitotic count of 2/50 HPF thus classified as a “low grade risk,” according to Miettinen's classification. The patient is currently disease-free, 8 months after the surgical resection.

**Case 2**

**Figure 3:**
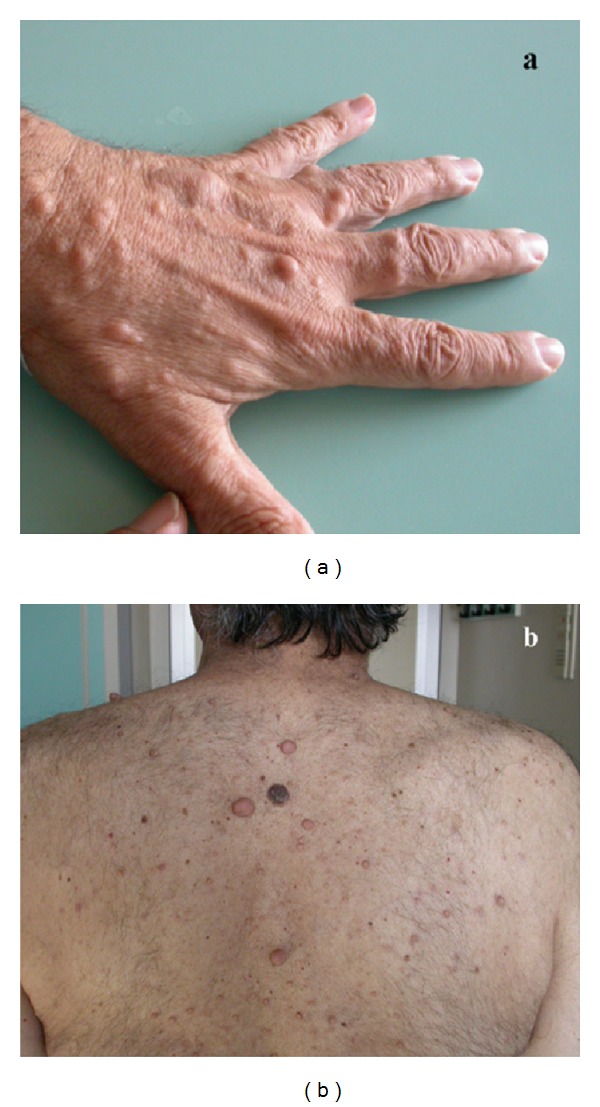
Cutaneous neurofibromas in patient affected by NF1 and duodenal GIST of the hand (a) and back (b).

A 56-year-old man was admitted to our department with a familial history of NF1 (Figure 3) and a past medical history consistent with a pheochromocytoma in 1994. The patient underwent a surgical resection elsewhere for a duodenal GIST (third portion) and presented to our attention with a relapse of the disease 7 months after the primary surgical resection. The patient was scheduled for a surgical procedure of duodenotomy and excision of the recurrence. The pathological description was consistent with a 4 cm c-KIT and CD34 positive GIST with 9 mitosis/50 HPF thus classified as at “intermediate risk” according to Miettinen's classification. The postoperative course was uneventful and the patient was discharged in 9th postoperative day. The patient is disease-free, 8 years after the surgical treatment.

### 3.2. Systematic Review

Table 1 summarizes the studies included in the present review of the literature. For the purpose of this investigation we pooled patients from different studies (23 articles, plus the present single centre experience), obtaining the clinical and pathological records of 252 GISTs detected in 126 NF1 patients [15–37].

As documented in Table 1, the vast majority of the studies reported single cases or small case series, excluding the article by Miettinen reporting 45 patients followed by the experience of Liegl and Andersson (resp., 16 and 15 NF1 patients) [18, 19, 27].

Table 2 shows the results of the data analysis. Patients were documented homogeneous regarding the M/F ratio (M/F 1), with a mean age at presentation of 52.8 years. Data regarding the NF1 syndrome (as a sporadic or familial disorder) has been detected exclusively in 17 out of the 126 NF1 included patients (13.5% of the series) and notably it has been documented as familial syndrome in the vast majority of the cases (70.6%). GISTs were reported as multiple tumors in the 35.3% of the patients and the prevalent localization was documented at the jejunum site 39.2%, followed by ileus in 30.6% of the cases. GISTs appeared to be incidental in the majority of the cases (52.5%) and were reported with a mean diameter of 3.8 cm. The mean mitotic count was documented to be 3.0/50 HPF and the pathological morphology was consistent with spindle-shaped cell tumors in almost the totality of the GISTs (93.0%). Indeed 97.4% were c-kit positive and 81.6% CD34 positive; moreover, even though data regarding the DOG-1 expression were available exclusively in 17 patients, 88.2% were reported as DOG-1 positive. Opposite anti-SMA antibodies were positive in 24.1% of the cases and S-100 protein was expressed in 30.3% of the tumors. Notably desmin expression has been documented negative in all the 109 tumors analyzed. Wild-type c-kit and PDGRF alpha genes were reported in 95.2% of the tumors analyzed for any mutations.

Of note, the 64.9% of the GISTs were reported as low risk tumors, otherwise the 17.5% were considered at intermediate risk and the 17.5% as high risk GISTs.

### 3.3. Comparison with Sporadic GISTs

Clinical and pathological features of GISTs associated with NF1 syndrome (including mean age at presentation, M/F ratio, tumors' localizations, symptoms at presentation, mean diameters, and risk classification) were compared for statistical purpose with those documented in sporadic GISTs in a subset of patients undergone surgical resection at our centre from 1999 to 2009 (n 47 patients) [38].  Table 3 summarizes results of the statistical analysis. We documented a difference of statistical value analyzing the mean age at presentation: indeed according to our results patients affected by NF1 were younger compared to sporadic GISTs patients (*t*-test *P* 0.0001). Moreover, in this subset of patients, tumors were significantly smaller (*t*-test *P* 0.0003). Tumors were located mainly in the jejunum/ileus in the NF1 subgroup whereas the main localization in the sporadic group was the stomach (Chi-square test *P* < 0.0001); moreover in the former group the vast majority of the GISTs were incidentally detected (Chi-square test *P* 0.002). Moreover, even though we did not document a significant difference analyzing the M/F ratios (Chi-square test *P* ns), we reported a prevalence of low-risk criteria in the NF1 subgroup compared with the sporadic GISTs (Chi-square test *P* 0.03).

## 4. Discussion

In this review we highlighted the clinical, pathological, and molecular features of GISTs detected in NF1 patients and, to the best of our knowledge, this is the first and most numerically relevant systematic analysis of the literature in this field. Moreover we reported our single centre experience regarding 2 GISTs detected in NF1 patients. Of note, from 1999 to date we treated 91 GISTs; thus the cases herein reported represent 2.2% of our series.

GISTs are commonly associated with NF1 syndrome, since a past study conducted on an autopsy series documented a GIST in one third of the NF1 patients [39].

GISTs associated with NF1 syndrome seem to have however a distinct phenotype: Miettinen reported that they occur in younger patients compared with sporadic GISTs that are often multiple and occur in the duodenum or small intestine [40].

Consistently with these findings, in our systematic review we highlighted that the mean age at presentation was 52.8 years and they are detected as multiple lesions in 35.3% of the cases, occurring in distal sites as the jejunum and small intestine.

Indeed in our previous research conducted on 47 primary GISTs patients, we reported a mean age at presentation of 61.4 years (median 62 years), none of the patients presented multiple lesions, and the most reported localization was the stomach, representing 59.6% of the tumors' sites [38].

Moreover, GISTs associated with NF1 patients have been described as clinically indolent and often asymptomatic, with low mitotic rates [40]. Indeed, we reported that 52.5% of the cases were described as incidental findings, whereas in our previous case series on GISTs, the patients were reported asymptomatic in 19.2% of the cases. Mean mitotic rate has been herein documented as 3/50 HPF; however also in our previous research 74.5% of the tumors had a mitotic count <5/50 HPF [38].

Notably a mutation in kit or PDGFR alpha genes has been reported exclusively in 4.8% of the cases.

Recently those GISTs associated with Carney Triad, Carney-Stratakis syndrome, along with young and pediatric GISTs have been documented to have a loss of succinate dehydrogenase subunit B (SDHB) expression, a mitochondrial protein [41–43]. On the basis of the SDHB expression, it has been recently proposed that GISTs could be differentiated into 2 characteristic subgroups: type 1 SDHB-positive and type 2 SDHB-negative [41]. Type 1 GISTs usually occur in adults with no predilection of tumor's locations, show homogeneous M/F, present KIT or PDGFRA mutations, and, generally, may benefit from the imatinib treatment. Type 2 GISTs occur usually in paediatric/young female patients almost exclusively in the stomach and present an epithelioid morphology. These tumors are usually c-kit and PDGFRa wild type and do not respond to the molecular treatment with imatinib [44].

Even though NF1 associated GISTs have been documented to be type 1 SDHB-positive tumors [43], they could be differentiated by several features including the predilection of localization to jejunum/small intestines, common tumor multiplicity, and the lack of GIST-specific mutations (kit and PDGFRa); moreover and alike the SDHB type 2 tumors, they do not respond well to imatinib treatment [43, 45, 46].

In conclusion, a wide review of the literature in this field documented that GISTs detected in NF1 patients seem to have a peculiar and distinct phenotype, different than the one commonly reported GISTs including younger age at presentation, distal localization, small diameter, low mitotic rate, and absence of kit and/or PDGRFa mutations. Moreover the vast majority of these tumors were documented to be kit positive consistent with spindle-shaped cells and were considered as low-risk neoplasms.

## Figures and Tables

**Table 1:** Systematic review of the literature from 2004 to date.

Reference	Author	Journal	Year	No. of patients	No. of GISTs
[15]	Cheng et al.	Digestive Diseases and Sciences	2004	3	5
[16]	Kinoshita et al.	The Journal of pathology	2004	7	29
[17]	Takazawa et al.	The American Journal of Surgical pathology	2005	9	36
[18]	Andersson et al.	The American Journal of Surgical pathology	2005	15	27
[19]	Miettinen et al.	The American Journal of Surgical pathology	2006	45	45
[20]	Maertens et al.	Human Molecular Genetics	2006	3	7
[21]	Nemoto et al.	Journal of Gastroenterology	2006	1	1
[22]	Bümming et al.	Scandinavian Journal of Gastroenterology	2006	1	4
[23]	Teramoto et al.	lnternational Journal of Urology	2007	2	2
[24]	Stewart et al.	Journal of Medical Genetics	2007	2	3
[25]	Kramer et al.	World Journal of Gastroenterology	2007	1	1
[26]	Invernizzi et al.	Tumori	2008	1	1
[27]	Liegl et al.	The American Journal of Surgical pathology	2009	16	16
[28]	Yamamoto et al.	Journal of Cancer Research and Clinical Oncology	2009	5	31
[29]	Dell'Avanzato et al.	Surgery Today	2009	1	2
[30]	Hirashima et al.	Surgery Today	2009	1	17
[31]	Cavallaro et al.	The American Journal of Surgery	2010	2	2
[32]	Relles et al.	Journal of Gastrointestinal Surgery	2010	2	5
[33]	Izquierdo and Bonastre	Anticancer Drugs	2012	1	4
[34]	Agaimy et al.	lnternational Journal of Clinical and Experimental pathology	2012	2	3
[35]	Ozcinar et al.	International Journal of Surgery Case Reports	2013	1	4
[36]	Vlenterie et al.	American Journal of medicine	2013	2	4
[37]	Sawalhi et al.	World Journal of Clinical Oncology	2013	1	1
		Present experience	2013	2	2

Total				126	252

**Table 2:** Clinical and pathological features from 252 GISTs in 126 NF1 patients.

Sex	*n*	%
M	59.0	50.0
F	59.0	50.0
Total available	**118.0**	**100.0**
Age (years)		
Mean; SD		52.8; 13
Range		19.0–82.0
Familial history	*n*	%
Sporadic	5.0	29.4
Familial	12.0	70.6
Total available	**17.0**	**100.0**
Number of GISTs	*n*	%
1	77.0	64.7
>1	42.0	35.3
Total available	**119.0**	**100.0**
Localization	*n*	%
Stomach	12.0	5.4
Duodenum	44.0	19.8
Jejunum	87.0	39.2
Ileus	68.0	30.6
Colon	4.0	1.8
Other	6.0	2.7
Not specified	1.0	0.5
Total available	**222.0**	**100.0**
Symptoms	*n*	%
Incidental	21.0	52.5
Bleeding	11.1	27.5
Pain	4.0	10.0
Palpable mass	4.0	10.0
Total available	**40.0**	**100.0**
GIST's diameter (cm)		
Mean		3.8; 4.3
Range		0.1–29.0
Mitotic index (*n*/50 HPF)		
Mean		3.0; 8.2
Range		0.0–57.0
Morphology	*n*	%
Spindle-shaped	159.0	93.0
Epithelioid	9.0	5.3
Mix	3.0	1.7
Total available	**171.0**	**100.0**
c-kit	*n*	%
Positive	151.0	97.4
Negative	4.0	2.6
Total available	**155.0**	**100.0**
CD34	*n*	%
Positive	84.0	81.6
Negative	19.0	18.4
Total available	**103.0**	**100.0**
Anti-SMA	*n*	%
Positive	19.0	24.1
Negative	60.0	75.9
Total available	**79.0**	**100.0**
S-100	*n*	%
Positive	33.0	30.3
Negative	76.0	69.7
Total available	**109.0**	**100.0**
Desmin	*n*	%
Positive	0.0	0,0
Negative	109.0	100.0
Total available	**109.0**	**100.0**
DOG-1	*n*	%
Positive	15.0	88.2
Negative	2.0	11.8
Total available	**17.0**	**100.0**
c-kit/PDGRFa Mutations	*n*	%
Presence	8.0	4.8
Absence—wild type	157.0	95.2
Total available	**165.0**	**100.0**
Risk classification	*n*	%
Low risk	37.0	64.9
Intermediate risk	10.0	17.5
High risk	10.0	17.5
Total available	**57.0**	**100.0**

**Table 3:** Comparison between GISTs presenting in NF1 patients and sporadic GISTs.

	GISTs in NF1 patients	GIST personal case series	*P* value
Age			
Mean (years)	52.8	61.4	0.0001*
Sex			
M/F ratio	1	1.61	0.2^§^
Localization (%)			
Stomach	5.4	56.9	<0.0001^§^
Jejunum/ileus	69.8	23.4	
Other	24.8	19.7	
Symptoms (%)			
Incidental	52.5	19.1	0.002^§^
Other	47.5	80.9	
Diameter			
Mean (cm)	3.8	7.4	0.0003*
Risk classification (%)			
Low risk	64.9	41.3	0.03^§^
Intermediate risk	17.5	21.7	
High risk	17.5	37.0	

*t-test; ^§^Chi-square test.
